# Adjuvant chemoradiotherapy does not improve outcomes in patients with fistula-associated anal adenocarcinoma undergoing abdominoperineal resection

**DOI:** 10.3389/fonc.2022.1061513

**Published:** 2022-11-09

**Authors:** Min Wang, Yu Xiang, Yunshan Wang, Jiayi Zhang, Haoran Zhao, Can Wang, Lichao Qiao, Bolin Yang

**Affiliations:** Department of Colorectal Surgery, Affiliated Hospital of Nanjing University of Chinese Medicine, Jiangsu Province Hospital of Chinese Medicine, Nanjing, China

**Keywords:** fistula-associated anal adenocarcinoma, abdominoperineal resection, chemoradiotherapy, treatment, literature review

## Abstract

**Objective:**

Abdominoperineal resection (APR) is currently established as a standard treatment regimen for fistula-associated anal adenocarcinoma (FAAA), however, the efficacy of chemoradiotherapy (CRT) remains unclear. The aim of this study is to evaluate the role of CRT in patients with FAAA treated with APR through single-center experience and literature review.

**Methods:**

A retrospective review was performed on patients with FAAA consecutive treated in our institution from 2005 to 2022. In addition, a systematic literature search was performed using PubMed and MEDLINE. All patients with FAAA who received APR in our institution and reported in the literature were included and divided into three categories for statistical analysis: APR alone (APR group), neoadjuvant therapy combined APR (CRT+APR group), and APR combined postoperative therapy (APR+CRT group).

**Results:**

Fifteen patients with FAAA were identified from our retrospective charts review. At a median follow-up time of 18 months, the recurrence-free survival rate was 53.3% and the survival rate was 73.3%. Eight patients underwent APR and 6 received postoperative chemotherapy. Among them, one died, one developed recurrence and the remaining six patients were alive with disease free. We found 37 publications describing 62 patients with FAAA treated with APR. Clinical data from these articles were analyzed together with the 8 cases in our institution. The overall survival rates were 94.1%, 70.8%, and 38.5% at 1-, 3-, 5-years respectively. Combining (neo)adjuvant therapy did not appear to improve outcomes in FAAA treated with APR (CRT+APR vs. APR, p=0.977; APR+CRT vs. APR, p=0.351). Lymph node involvement was shown to be significantly associated with poor outcomes by multivariate analysis (p=0.020).

**Conclusions:**

For patients with FAAA without lymph node involvement, APR is adequate to control disease and the addition of CRT does not appear to prolong survival.

## Introduction

Fistula-associated anal adenocarcinoma (FAAA) is a rare malignant transformation of anal fistula, which develops in 0.1% of all anal fistula as reported (1). In 1934, Rosser first described this malignancy, and established a clinical diagnostic criteria: 1) the fistula should usually antedate the carcinoma by at least 10 years, 2) the only tumor present in the rectum or anal canal should be secondary to direct extension from the carcinoma in the fistula, and 3) the internal opening of the fistula should be into the anal canal and not into the tumor itself (2). Though this tumor has been increasingly reported recently, most of the studies were case reports or small case series. The lack of large sample data makes it difficult to establish a standard regimen for the treatment of this tumor.

Surgery is the mainstay treatment for resectable tumors, and abdominoperineal resection (APR) is currently established as a standard surgical procedure for FAAA (3, 4). It was reported that the 5-year survival rates of anal adenocarcinoma were significantly better in the surgery group compared to the nonoperative group (50-58% and 30%, respectively, p=0.03) (5). In addition, patients with radical resection had better 5-year survival rates than those treated with non-radical resection (6).

The role of chemoradiotherapy (CRT) in FAAA is still controversial. Some authors believe that CRT especially neoadjuvant therapy can improve prognosis of FAAA (5, 7), though the survival benefit of CRT has not been specifically documented in any high-quality random trial. Some authors believe that APR with negative surgical margins appears to be sufficient, additional CRT will not give any positive outcome but only delay the definite surgical management (8, 9). The rationale for selecting CRT is still weak and further studies are required to establish the optimal management in this uncommon malignancy.

This study aimed to explore the role of CRT in patients with FAAA treated with APR through a single-center retrospective analysis and an overview of the literature.

## Materials and methods

We searched the Electronic Medical Records System of the Jiangsu Province Hospital of Chinese Medicine (Nanjing, China) for all cases of anal fistula and anal adenocarcinoma occurring between 1 July 2005 and 31 March 2022. Cases with anal adenocarcinoma not associated with anal fistula were excluded. Collected information included: age, gender, demographics, clinical presentation, therapy, and outcome. Informed consent was exempted by the hospital ethical committee due to the retrospective nature of the study.

### Systematic Review

A bibliographical search was performed of PubMed and MEDLINE from October 1952 to December 2020. The keywords used were: ‘perianal fistula’, ‘anal fistula’, ‘adenocarcinoma’, and ‘mucinous adenocarcinoma’, either singly or in combination. The reference lists from relevant articles were also searched for additional studies. The inclusion criteria included the following: 1) carcinomas were associated with anal fistula; 2) patients who received APR; 3) the study was reported in English. Exclusion criteria included the following: 1) rectal adenocarcinoma without a history of anal fistula; 2) patients received CRT alone or refused any therapy; 3) studies not reporting length or results of follow-up; 4) overlapping studies. In the case of duplicate publications, the most informative article was chosen. All patients with FAAA who received APR in our institution and reported in the literature were included and divided into three categories for statistical analysis: APR alone (APR group), neoadjuvant therapy combined APR (CRT+APR group), and APR combined postoperative therapy (APR+CRT group).

### Statistical Analysis

Continuous variables were described as mean with standard deviation (SD) or median values with interquartile ranges (IQR). Categorical variables were expressed as number (percentage). Kaplan-Meier curves were used to calculate cumulative probability of survival. The log-rank test was applied to compare the difference in survival. Multivariate analysis of survival was performed using the Cox proportional hazards model. All statistical analyses and graphical representations were performed using SPSS 25.0 (SPSS, Chicago, Illinois, USA) and Graph Pad Prism 9 (Graph Pad Software, San Diego, USA) software. A p-value <0.05 was considered statistically significant.

## Results

A total of fifteen patients with FAAA (1 female and 14 males, mean age 54.5 years) were found at our institution, represented 0.17% of the 8823 patients undergoing surgery for anal fistula at our institution from July 2005 to March 2022 (**Table 1**). All these 15 patients had chronic anal fistulas, with a median duration of 5 (range, 0.75-30) years. One patient had a history of Hirschsprung’s disease. Two patients had Crohn’s disease and one of them underwent terminal ileal resection. Prior to the diagnosis of FAAA, a total of 24 operations for anal fistula or abscess were performed on 11 (11/15, 73.3%) patients. Surgical specimens were evaluated routinely in previous anal fistula surgery, and no malignant change was reported.

**Table 1:** Fistula-associated anal adenocarcinoma in 15 patients.

Patient	Gender	Age (y)	Fistula duration (y)	Histological type	Stage	Treatment	Time of follow-up (months)	Outcome
1	M	56	20	AD	T3N1M0	APR + postoperative chemotherapy	18	Death
2	F	52	2	AD	T2N0M0	APR	6	Alive
3	M	41	10	MA	T2N0M0	APR + postoperative chemotherapy	17	Alive
4	M	58	10	AD	T2N2M0	CRT	11	Recurrence
5	M	47	0.75	MA	T3N0M0	APR + postoperative chemotherapy	18	Alive
6	M	32	3.75	MA	T3N0M0	APR + postoperative chemotherapy	15	Recurrence
7	M	55	2	MA	T2N0M0	APR + postoperative chemotherapy	44	Alive
8	M	52	15	MA	T3N0M0	CRT	36	Death
9	M	59	3	MA	T3N0M0	CRT	30	Death
10	M	61	12	MA	T3N2M0	No treatment	6	Death
11	M	65	5	MA	T3N0M0	Local resection + CRT	74	Alive
12	M	72	30	MA	T2N0M0	APR	47	Alive
13	M	55	15	MA	T4N3M1	Palliative colostomy	1	Alive
14	M	61	1	MA	T3N2M0	On NACRT	5	Alive
15	M	51	2	MA	T2N0M0	APR + postoperative chemotherapy	1	Alive

M, male; F, female; AD, adenocarcinoma; MA, mucinous adenocarcinoma; APR, abdominoperineal resection; CRT, chemoradiotherapy; NACRT, neoadjuvant chemoradiotherapy.

All the cases were histologically proven by biopsy or tumor resection, including 3 cases of adenocarcinoma and 12 cases of mucinous adenocarcinoma. Nine (9/15, 60.0%) patients had T3-T4 tumors. Five (5/15, 33.3%) had lymph node involvement, including one with bone metastasis. Six patients presented with elevated levels of carcinoembryonic antigen or carbohydrate antigen 19-9. All patients underwent colonoscopy at the same time of diagnosis, one had a tubular adenoma of the transverse colon and one had a rectal tubulovillous adenoma of the rectum with moderate dysplasia.

**Figure 1:**
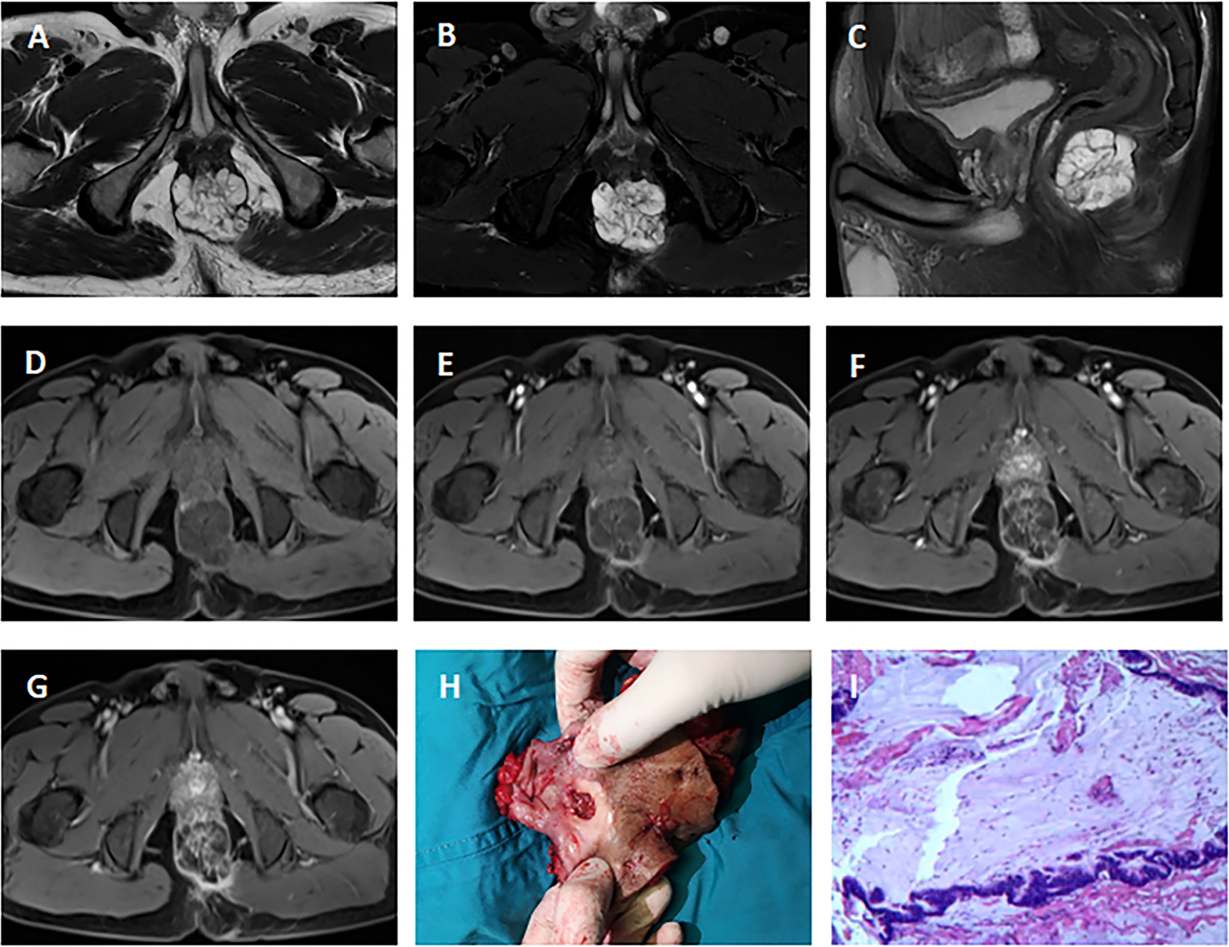
MRI of a 51-year-old man with mucinous adenocarcinoma. **(A)**: Axial T2-weighted image; **(B)**: Axial fat-suppressed T2-weighted image; **(C)**: Sagittal fat-suppressed T2-weighted image; **(D–G)**: Axial dynamic contrast-enhanced images. The tumor grew across the midline. This lesion was multiloculated, cauliflower-like, markedly hyperintense on fat-suppressed T2-weighted image. Axial dynamic contrast-enhanced images showed progressive heterogeneous enhancement, and the boundary of the enhanced region was clear. **(H)**: Clinical photograph showed the abdominoperineal resection specimen (the proximal intestinal canal was cut up). **(I)**: The tumor was rich in mucus (hematoxylin and eosin staining; original magnification, ×100).

Pelvic MRI was performed in all 15 cases. Among the patients with adenocarcinoma, MRI scan demonstrated heterogeneous solid lesions on T2-weighted images, and heterogeneous enhancement on contrast enhanced sequences. In patients with mucinous adenocarcinoma, tumors were multiloculated and cauliflower-like, appeared as markedly hyperintense on fat-suppression T2-weighted images. Contrast enhanced MR sequences demonstrated progressive heterogeneous enhancement that was more evident in the periphery of the tumor and less intense within the central areas of higher mucin content (**Figure 1**). Compared with adenocarcinoma, mucinous adenocarcinoma demonstrated lower signal intensity on diffusion-weighted imaging and higher signal intensity on the apparent diffusion coefficient map. In general, if the short axis diameter of a lymph node is >5mm, morphological change (irregular or round), hyperintense on fat-suppression T2-weighted images, heterogeneous enhancement on contrast-enhanced images, then metastasis in this lymph node is suspected (10). There were five patients in our group showing suspected lymph node metastasis (one involved the perirectal lymph nodes, four involved iliac lymph nodes or inguinal lymph nodes), and three of them obtained pathologic confirmation.

A total of 8 patients (53%) received APR, and 6 of these received postoperative chemotherapy (FOLFOX-6). R0 resection was achieved in all patients undergoing radical surgery, but the lateral margin was <1mm in one patient (who recurred at 16 months and died 2 months later from distant metastasis). The remaining 7 patients were alive during the follow-up period, and one of them recurred at 15 months. Seven patients did not receive radical surgery. One declined any intervention, 3 received CRT alone, and one underwent local resection combined with CRT. All these five patients experienced disease progression (including recurrence or metastasis) during the follow-up, and 3 of them died from distant metastasis. The rest two patients were diagnosed newly, one underwent a palliative colostomy because of severe local and metastatic disease, the other one is on neoadjuvant therapy at present.

**Figure 2:**
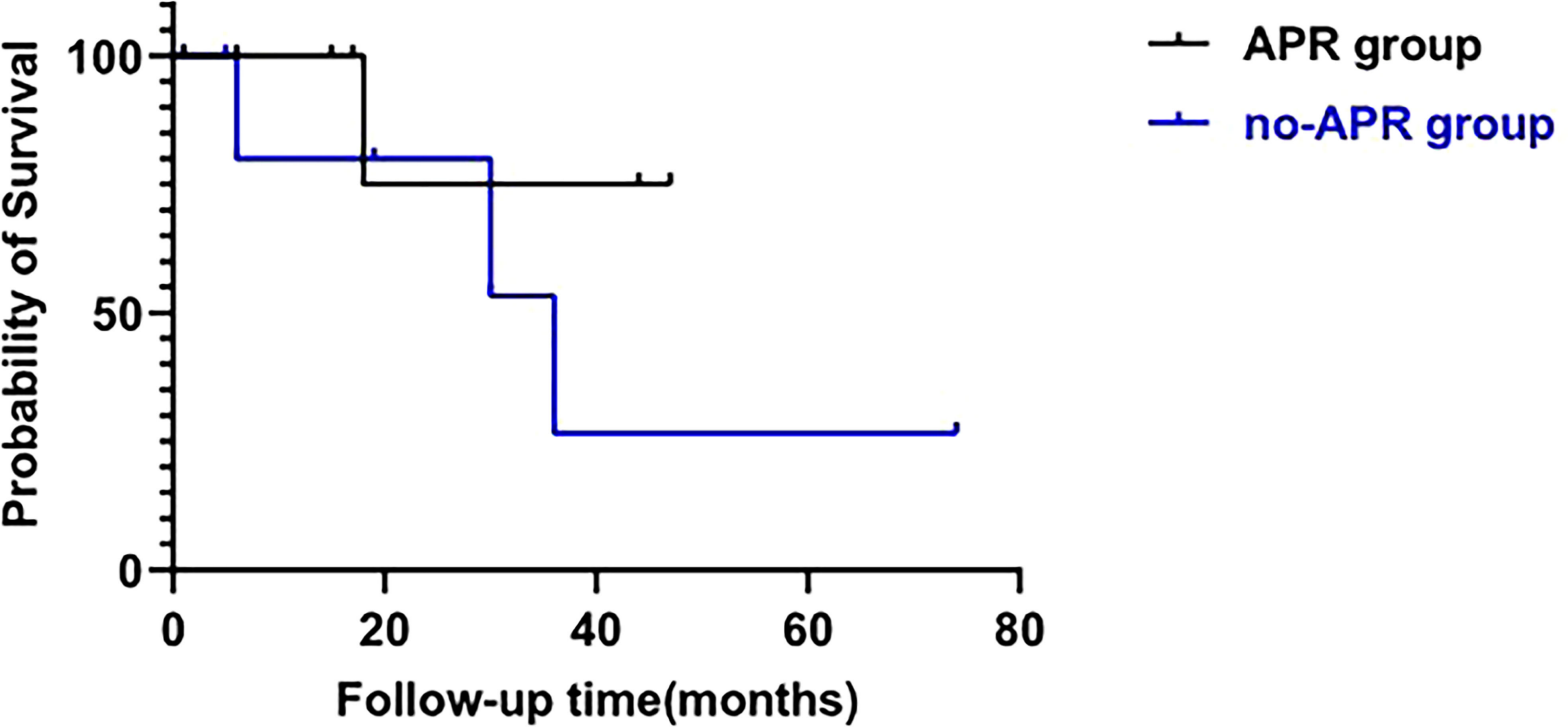
Survival analysis of patients treated with APR (APR group) and patients who did not receive APR (no-APR group). (log-rank test; p=0.321).

In general, with a median follow-up of 18 (IQR, 6-33) months, the recurrence-free survival rate was 53.3% and the overall survival rate was 73.3%. The overall survival rate for those who did not receive APR was 57.1% compared with 87.5% for those who underwent APR (**Figure 2**).

### Systematic review

Sixty-two patients from 37 studies were finally included in the systematic review (8, 11–46). Clinical data from these articles were pooled with the 8 cases from our institution. The final analyses included 70 patients (11 female, mean age 56.6 years). The mean duration of anal fistula was 13.7 (range, 0.17-56) years, and nearly half of the patients had a duration of anal fistula shorter than 10 years. There were 52 (74.3%) patients with mucinous adenocarcinoma and 18 (25.7%) with adenocarcinoma. The TNM classification was T2 in 19.1%, T3 in 61.8% and T4 in 19.1%. At time of diagnosis, lymph node involvement was documented in 13 patients (22.0%).

The population was divided into three groups according to treatment modality. Thirty-four patients (48.6%) underwent APR alone (APR group), 19 patients (27.1%) received neoadjuvant therapy combined APR (CRT+APR group), and 17 patients (24.3%) received APR combined postoperative therapy (APR+CRT group). Most patients (12/13, 92.3%) with lymph node involvement received APR combined (neo)adjuvant therapy. The patients’ clinical characteristics were summarized in **Table 2**.

**Table 2:** Systematic review: Patient characteristics (n=70).

Characteristic	Study cohort (n=70)	APR group (n=34)	CRT+APR group (n=19)	APR+CRT group (n=17)
Age (y)				
Median	56.0	58.5	62.0	47.0
Mean ± SD	56.6 ± 12.1	57.5 ± 12.1	60.4 ± 11.1	50.5 ± 11.5
Range	32.0-80.0	35.0-79.0	34.0-80.0	32.0-80.0
Gender				
Female	11 (16)	5 (15)	4 (21)	2 (12)
Male	59 (84)	29 (85)	15 (79)	15 (88)
Fistula duration (y)				
<10	28 (40)	13 (38)	8 (42)	7 (41)
≥10	31 (44)	18 (53)	6 (32)	7 (41)
ND	11 (16)	3 (9)	5 (26)	3 (18)
Crohn’s disease				
No	54 (77)	28 (82)	16 (84)	10 (59)
Yes	16 (23)	6 (18)	3 (16)	7 (41)
Histological type				
AD	18 (26)	13 (38)	3 (16)	2 (12)
MA	52 (74)	21 (62)	16 (84)	15 (88)
T stage				
T2	9 (13)	4 (12)	1 (5)	4 (23)
T3	29 (41)	8 (23)	11 (58)	10 (59)
T4	9 (13)	4 (12)	2 (11)	3 (18)
ND	23 (33)	18 (53)	5 (26)	0 (0)
N stage				
N0	46 (66)	27 (79)	8 (42)	11 (65)
N1/2/3	13 (18)	1 (3)	6 (32)	6 (35)
ND	11 (16)	6 (18)	5 (26)	0 (0)
M stage				
M0	49 (70)	21 (62)	11 (58)	17 (100)
M1	0 (0)	0 (0)	0 (0)	0 (0)
ND	21 (30)	13 (38)	8 (42)	0 (0)
Follow up time (months)	24 (24)	25 (35)	24 (16)	16 (22)

APR, abdominoperineal resection; CRT, chemoradiotherapy; SD, standard deviation; AD, adenocarcinoma; MA, mucinous adenocarcinoma; ND, not defined.

All values expressed as n (%) or median (IQR), unless otherwise noted.

**Figure 3:**
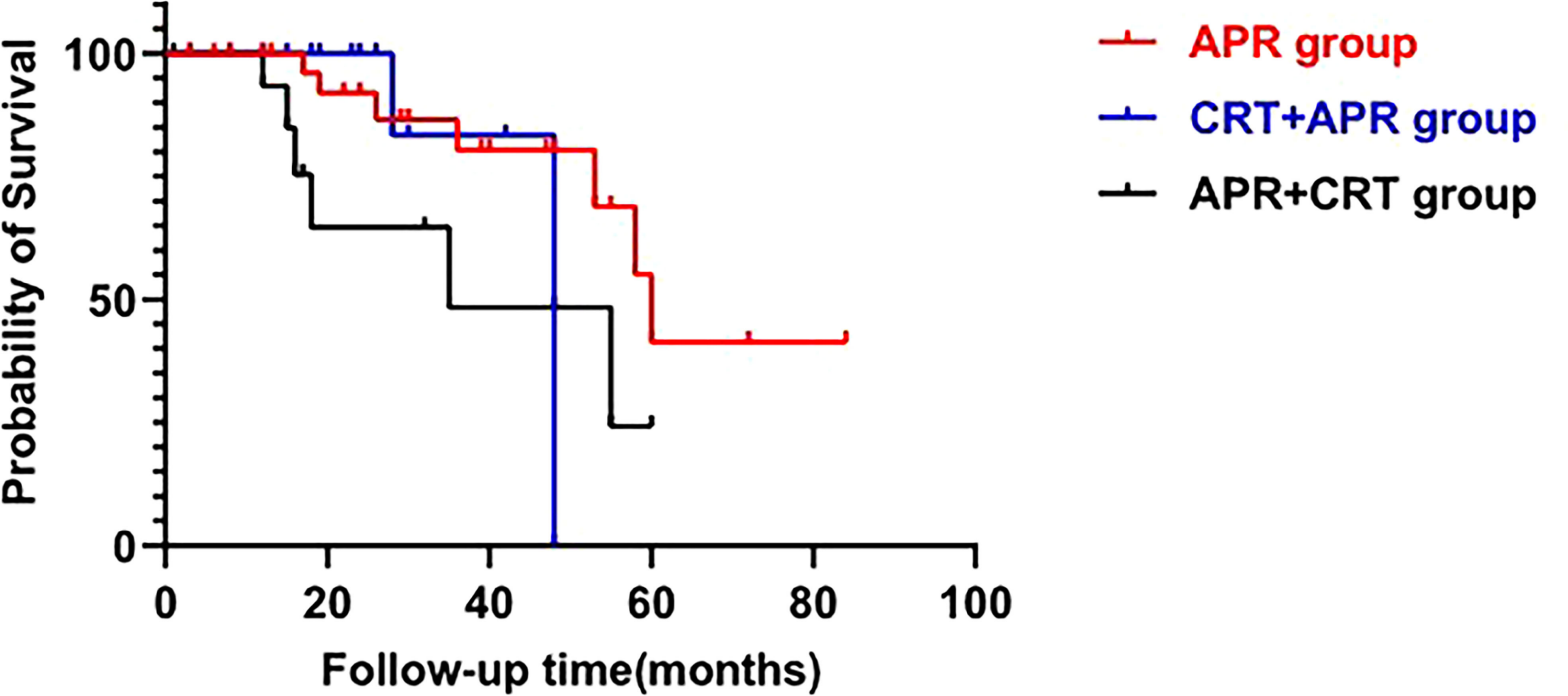
Overall survival across three treatment modalities. (log-rank test; p=0.616).

Tumor recurrence was documented in 19 of 70 cases (27.1%), among them, 14 patients died of tumor progression at a median follow-up of 19 (IQR, 15-33) months. Moreover, 3 patients died of non-oncological disease during follow-up. Median overall survival was 58 months (95%CI: 49.20-66.81), with 60 months in the APR group, 48 months in the CRT+APR group, and 35 months in the APR+CRT group (p=0.616, **Figure 3**). The 1-, 3-, and 5-year overall survival rates were 94.1%, 70.8%, and 38.5%, respectively. The addition of CRT did not appear to improve outcomes in patients treated with APR (CRT+APR vs. APR, p=0.977; APR+CRT vs. APR, p=0.351). In patients with N0 stage, the result also showed that the addition of CRT cannot prolong survival (p=0.355).

**Figure 4:**
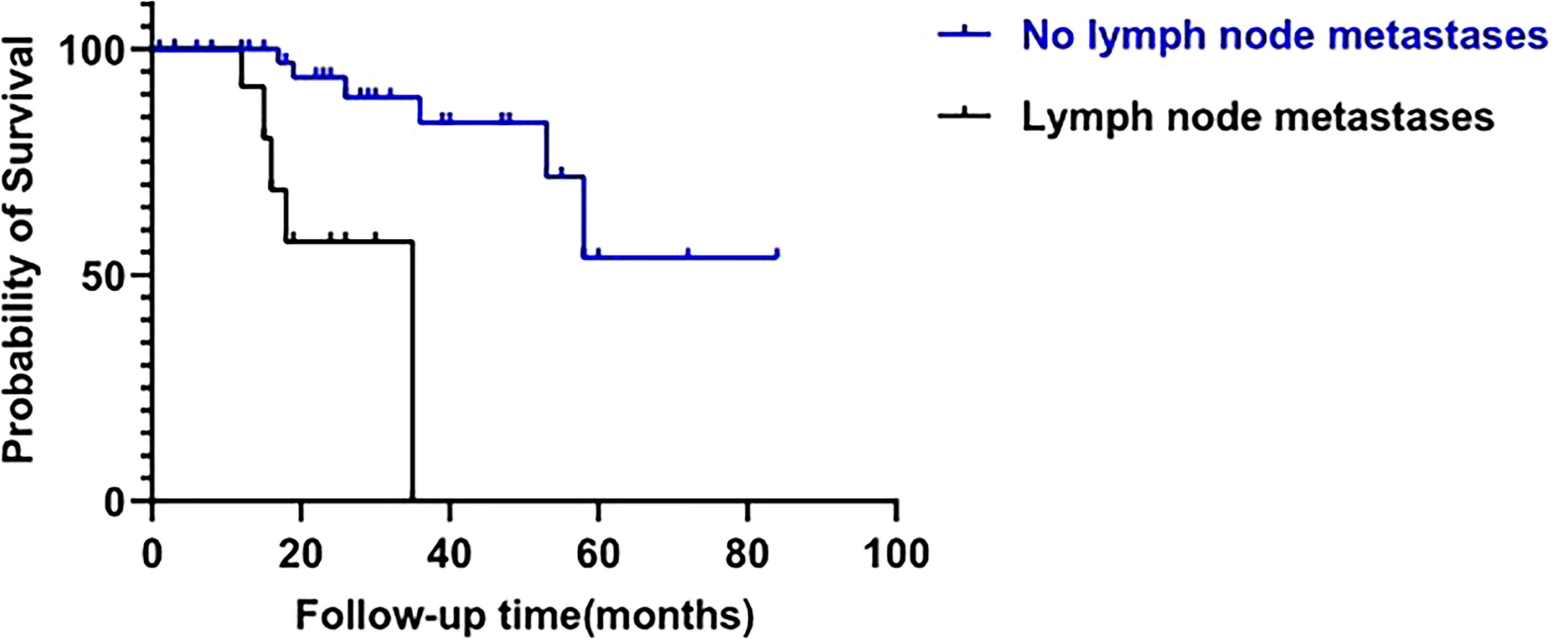
Impact of N stage on overall survival of fistula-associated anal adenocarcinoma. (log-rank test; p=0.001).

Univariate and multivariate analyses were performed to investigate the prognostic factors for survival of patients with FAAA. Lymph node involvement was shown to be significantly associated with poor outcomes by univariate analysis (p=0.001, **Figure 4**), and continued to be an independent negative prognostic factor for overall survival by multivariate cox analysis (HR=22.10, 95%CI: 1.64-298.36, p=0.020). Five of 13 patients (38.5%) with positive lymph node involvement died of recurrence or metastasis. Among the 46 patients without lymph node involvement, only 7 (15.2%) died during follow up.

## Discussion

Due to the rarity of FAAA and the lack of sufficient patients for controlled trials, there is still no consensus regarding treatment strategies. Whether the use of CRT would result in longer survival remains uncertain. This study addresses the role of CRT in patients with FAAA treated with APR. Our results show that APR is sufficient for patients with no lymph node involvement, and the additional CRT cannot improve prognosis.

In our cohort, in addition to two newly diagnosed patients, five patients did not receive APR because the lesions were judged to be unresectable or the patients rejected to undergo radical surgery. Four of five patients who did not receive APR developed disease progression within three years, and the remaining one who underwent local resection combined with CRT relapsed at 74 months post-operatively. Patients who underwent APR had better survival rate than patients who did not receive APR (87.5% and 57.1%, respectively). As only 8 of 15 patients received APR, and 6 of these received postoperative chemotherapy, the role of CRT in patients treated with APR cannot be well elucidated. To further analyze this problem, we performed a systematic literature review and found that (neo)adjuvant CRT cannot prolong survival of FAAA treated with APR (CRT+APR vs. APR, p=0.977; APR+CRT vs. APR, p=0.351). Notably in our study, most patients (12/13, 92.3%) with lymph node involvement received APR combined CRT. In patients with N0 stage, the overall survival rate was similar whether combined CRT or not (p=0.355). It means that treatment regimens should be chosen according to the disease stage. For patients without lymph node metastasis, APR is sufficient and there is no requirement for CRT.

Given the overall lack of experience and limited descriptions in the literature, the management of FAAA is advised tends to follow that of rectal adenocarcinoma (47). As suggested by some authors, CRT may play an important role in the treatment of FAAA, APR combined (neo)adjuvant therapy may be a reasonable treatment approach (29). Gaertner et al. reported 11 patients with FAAA who received APR, in 7 of these after neoadjuvant chemoradiotherapy (NACRT). All patients receiving NACRT combined APR were alive with disease free, whereas 2 of 4 patients without NACRT had died of tumor progression during the follow-up (48). However, anal adenocarcinoma and rectal cancer are identified as different entities. Anal adenocarcinoma differs from rectal disease which responds well to CRT (9). In addition, mucinous tumors respond more poorly to CRT than non-mucinous tumors (49). The view had been that mucinous tumors show little response to adjuvant therapy, and mucinous adenocarcinoma is in fact the most common type of FAAA. In conclusion, the rationale for selecting either neoadjuvant, adjuvant, or combined therapies is still weak. Further studies comparing the oncologic outcome of patients with FAAA undergoing radical surgery with or without (neo)adjuvant CRT are warranted to evaluate the role of CRT.

Previous studies have suggested that lymph node metastasis is associated with poor prognosis in FAAA (25). Our study showed similar findings. We had demonstrated an increased risk for cancer-related death in patients of FAAA with lymph node involvement. Preoperative MRI is of the utmost importance for predicting lymph node metastasis. A high suspicion of lymph node metastasis is issued in case of the presence of mixed signal intensity and/or an irregular border of the nodal capsule, irrespective of nodal size (50). NACRT combined with surgery may be an effective treatment approach to prolong the survival when lymph node metastasis is suspected prior to surgery. It was reported that NACRT can induce tumor regression, downstaging, cause downsizing of large and advanced tumors, thus increasing the rate of R0 resection and decreasing the incidence of local recurrence (4, 27, 42). However, routine delivery of NACRT to all patients with imaging predicted lymph node metastasis remains controversial in view of the lack of prognostic relevance of the preoperative MRI assessment of involved lymph nodes on the risk of local recurrence. Further, when lymph node metastasis is proven after surgery, adjuvant CRT should be considered, though more data supporting this recommendation are required.

The incidence of FAAA seems related to the duration of anal fistula. In the past, it was generally believed that FAAA occurs in patients with at least a 10-year history of perianal disease (48, 51). However, nearly half of the patients in our systematic review had a duration of anal fistula shorter than 10 years. This issue deserves special attention by clinicians as it implies the progression from a benign anal fistula to FAAA may be faster than previously described. A high index of suspicion must be maintained in patients with recurrent or incurable anal fistulas regardless of the duration of fistula, especially when patients presented gelatinous material or palpable mass (29, 52). For patients with highly suspicious symptoms, earlier monitor should be performed to avoid missed diagnosis and misdiagnosis.

The present study has several limitations. First, this was a retrospective study, and the investigations included were based on small sample size in their reports. The results may therefore not be entirely accurate. Second, the variety of treatment modalities for FAAA described in the literature and the lack of consistency in CRT regimens may limit definitive conclusions.

## Conclusion

We hold the opinion that APR is sufficient for FAAA at an early stage. When lymph node metastasis is suspected on preoperative MRI, or is pathologically proved after the operation, the combining of CRT may be a therapeutic option. The long-term survival rate of FAAA is poor, large-scale trials should be performed to establish the optimal therapeutic strategy of this tumor further.

## Author contributions

BY conceived the study and participated in the study design and performance. MW and YX extracted the data and drafted the manuscript. YW, JZ, HZ, CW and LQ revised the manuscript. All authors have read and agreed to the published version of the manuscript.

## Funding

This study was supported by Jiangsu Province Hospital of Chinese Medicine Peak Talent Program (y2021rc27).

## Acknowledgments

We would like to extend our gratitude to the researchers and study patients for their contributions.

## Conflict of interest

The authors declare that the research was conducted in the absence of any commercial or financial relationships that could be construed as a potential conflict of interest.

## Publisher’s note

All claims expressed in this article are solely those of the authors and do not necessarily represent those of their affiliated organizations, or those of the publisher, the editors and the reviewers. Any product that may be evaluated in this article, or claim that may be made by its manufacturer, is not guaranteed or endorsed by the publisher.
